# Development of Fast and Portable Frequency Magnetic Mixing-Based Serological SARS-CoV-2-Specific Antibody Detection Assay

**DOI:** 10.3389/fmicb.2021.643275

**Published:** 2021-05-05

**Authors:** Jan Pietschmann, Nadja Voepel, Leonie Voß, Stefan Rasche, Max Schubert, Michael Kleines, Hans-Joachim Krause, Tamlyn M. Shaw, Holger Spiegel, Florian Schroeper

**Affiliations:** ^1^Fraunhofer Institute for Molecular Biology and Applied Ecology IME, Aachen, Germany; ^2^Laboratory Diagnostic Center, University Hospital RWTH Aachen, Aachen, Germany; ^3^Institute of Biological Information Processing, Bioelectronics (IBI-3), Forschungszentrum Jülich, Jülich, Germany; ^4^Cape Biologix Technologies, Cape Town, South Africa

**Keywords:** COVID-19, immunofiltration, magnetic beads, magnetic immunodetection, *Nicotiana benthamiana*, Point of care diagnostic, recombinant S1 antigen production, transient expression

## Abstract

A novel severe acute respiratory syndrome coronavirus-2 (SARS-CoV-2) emerged in China in December 2019, causing an ongoing, rapidly spreading global pandemic. Worldwide, vaccination is now expected to provide containment of the novel virus, resulting in an antibody-mediated immunity. To verify this, serological antibody assays qualitatively as well as quantitatively depicting the amount of generated antibodies are of great importance. Currently available test methods are either laboratory based or do not have the ability to indicate an estimation about the immune response. To overcome this, a novel and rapid serological magnetic immunodetection (MID) point-of-care (PoC) assay was developed, with sensitivity and specificity comparable to laboratory-based DiaSorin Liaison SARS-CoV-2 S1/S2 IgG assay. To specifically enrich human antibodies against SARS-CoV-2 in immunofiltration columns (IFCs) from patient sera, a SARS-CoV-2 S1 antigen was transiently produced in plants, purified and immobilized on the IFC. Then, an IgG-specific secondary antibody could bind to the retained antibodies, which was finally labeled using superparamagnetic nanoparticles. Based on frequency magnetic mixing technology (FMMD), the magnetic particles enriched in IFC were detected using a portable FMMD device. The obtained measurement signal correlates with the amount of SARS-CoV-2-specific antibodies in the sera, which could be demonstrated by titer determination. In this study, a MID-based assay could be developed, giving qualitative as well as semiquantitative results of SARS-CoV-2-specific antibody levels in patient’s sera within 21 min of assay time with a sensitivity of 97% and a specificity of 92%, based on the analysis of 170 sera from hospitalized patients that were tested using an Food and Drug Administration (FDA)-certified chemiluminescence assay.

## Introduction

Since its discovery in Wuhan China in December 2019, the novel severe acute respiratory syndrome coronavirus-2 (SARS-CoV-2) has developed into an ongoing global pandemic, with more than 117 million cases (while writing this article), daily increasing numbers, and a global death number of more than 2.6 million deaths (Dong et al., 2020; Zhou et al., 2020; Zhu N. et al., 2020). The clinical manifestation of the so-called coronavirus disease 2019 (COVID-19) in infected individuals ranges from mild cold-like symptoms towards severe etiopathology including lung failure and death especially in elderly patients (Li et al., 2020; Wang D. et al., 2020; Zhu J. et al., 2020). However, up to 45% of SARS-CoV-2-infected patients experience an asymptomatic but still infective disease process, thus representing a major source for virus transmission within populations through droplet infection (Kolifarhood et al., 2020; Nikolich-Zugich et al., 2020; Oran and Topol, 2020). To prevent a completely uncontrolled spread, almost worldwide nonpharmaceutical restrictions have been imposed to contain the existing extremely high infection rate of the virus. However, long-lasting control of the SARS-CoV-2 pandemic appears to be achievable only by generating population’s immunity. Currently, there are at least 74 different vaccine candidates in different stages of clinical development, and some have shown more than 90% efficacy against SARS-CoV-2 infection in Phase III trials (Zimmer et al., 2020; Knoll and Wonodi, 2021). Based on accelerated approval from regulatory authorities, the first vaccination programs have already been initiated, spearheading upcoming global vaccination campaigns. Especially in the early phase of the vaccine campaigns, the monitoring of SARS-CoV-2-specific antibody responses in vaccinated individuals is essential to confirm the ability of the vaccine to induce sufficient antibody titers to protect from disease and infection and, if possible, provide sterile immunity. Since the global demand for vaccine doses massively exceed the production capacities, it is necessary to prioritize vaccine distribution. Identifying individuals that already have SARS-CoV-2-specific antibodies will enable more targeted vaccine campaigns. The availability of cheap and reliable, easy-to-use solutions for the determination of antibody titers in human sera will be a key issue in this context.

Currently, several FDA-approved analytical methods can be used for the detection of SARS-CoV-2-specific antibodies in serological specimens. For most assays, the SARS-CoV-2 Spike glycoprotein (S), comprising of the S1 and S2 subunit, is being used since it allows the reliable detection of SARS-CoV-2-specific antibody responses. It is a known target of neutralizing antibodies that prevents virus entry through the inhibition of the interaction of the virus with the host cell receptor angiotensin-converting enzyme 2 (ACE2) (Krüttgen et al., 2020; Walls et al., 2020; Zhao et al., 2020). In contrast to the other viral antigens, the envelope protein (E), membrane protein (M), the nucleocapsid protein (N), or hemagglutinin esterase (HE), which show significant homologies with other known and previously circulating coronaviruses (hCoV), such as hCoV-229E, hCoV-NL63, hCoV-OC43, and hCoV-HKU1, or SARS-CoV and Middle East respiratory syndrome coronavirus (MERS-CoV), SARS-CoV-2 S-protein-derived specific antibodies appear to be highly species specific (Agnihothram et al., 2014; Khan et al., 2020).

The market provides lateral flow detection (LFD)-based solution to quick-check serum samples for SARS-CoV-2-specific antibodies. These assays are fast (10–20 min assay duration) but not suitable for the quantification of antibody titers since the principle provides simple colorimetric yes/no answers (qualitative answer). Although currently 17 LFD-based assays are approved for the qualitative analysis of serum samples by the FDA for the detection of human IgG, IgM, or both, a sensitive and quantitative analysis can only be done using laboratory-based methods as enzyme-linked immunosorbent assays (ELISAs) or chemiluminescence-based assays (FDA – U.S. Federal Drug Administration, 2020). Both are currently irreplaceable but not suitable for easy on-site testing of serological samples since large and sensitive benchtop instruments are required for the readout. Rapid and sensitive point-of-care (PoC) applicable assays to qualitatively and quantitatively assess SARS-CoV-2-specific antibodies are currently not available.

Here, magnetic immunodetection (MID) based on sensing superparamagnetic particles by frequency magnetic mixing detection (FMMD) technology might be a powerful tool. The implementation of MID-based assays for the detection of various antigens like bacterial and fungal toxins, plant and human pathogens, as well as peptide-specific antibodies was previously demonstrated (Meyer et al., 2007a,b,c; Rettcher et al., 2015; Achtsnicht et al., 2019a; Pietschmann et al., 2020a,b,c. The basic principle of magnetic immunodetection is the specific enrichment of an analyte within an immunofiltration column (IFC) and its subsequent labeling with functionalized magnetic nanoparticles, resulting in an analyte dose-dependent retention of particles. Subsequently a particle-dose-depending measuring signal in millivolt (mV) is obtained, using a portable measuring FMMD device (Krause et al., 2007; Achtsnicht et al., 2019a,c; Pietschmann et al., 2020a,b,c. With this, a correlation between antigen and measuring signal can be seen, resulting not only in a qualitative but also quantitative data. A detailed and complete description of FMMD can be found elsewhere (Krause et al., 2007; Pietschmann et al., 2020a.

Based on previously described drawbacks of currently used analytical PoC methods, MID can be a great tool for rapid on-site SARS-CoV-2 antibody detection in combination with antibody titer determination. For the development of serological MID, we analyzed in total 170 human sera samples from hospitalized patients using the FDA-approved laboratory-based Liaison SARS-CoV-2 S1/S2 IgG assay for detecting virus-specific antibodies. Simultaneously, we used a transient plant-based expression system to produce recombinant SARS-CoV-2 S1 antigen for specific enrichment of antibodies within the IFC. Afterward, the sera were used for verification of sensitivity as well as specificity using our optimized MID-based quantitative and qualitative detection assay. Here, we demonstrate the development of a rapid MID-based PoC assay enabling a highly sensitive, specific, and semiquantitative detection of SARS-CoV-2-specific antibodies in human serum within 21 min.

## Materials and Methods

### Ethics Statement

A total of 170 sera of patients with COVID-19-like symptoms hospitalized in the University Hospital RWTH Aachen, Germany, were included into this study. Sample and data acquisition were approved by the Medical Ethics Committee of the University Hospital RWTH Aachen (EK 093/2020).

### Transient Transformation of *N. benthamiana*

Recombinant His6-tagged SARS-CoV-2 S1 protein was produced in *Nicotiana benthamiana* plants by transient transfection. *Agrobacterium tumefaciens* strain GV3101::pMP90RK (CarbR, KmR, RifR) was transformed with a SARS-CoV-2 S1-His protein encoding binary expression vector provided by collaborators Cape Biologix Technologies, Cape Town, South Africa. *N. benthamiana* plants were infiltrated as described previously (Koncz and Schell, 1986; Rademacher et al., 2008; Simmons et al., 2009).

After cultivation of *A. tumefaciens* for 48 h (28°C, 160 rpm) in peptone *Agrobacterium* medium (PAM) (Houdelet et al., 2017) supplemented with 25 μg ml^–1^ kanamycin and 50 μg ml^–1^ carbenicillin, the infiltration broth was prepared by diluting the culture with 2 × infiltration media [10% (*w/v*) sucrose, 0.4% (*w/v*) glucose, 1% (*w/v*) Ferty II mega, pH 5.6] and water to OD_600_ = 1. To improve recombinant protein yields, the final broth was mixed 1:5 with *A. tumefaciens* culture (OD_600_ = 1) harboring a binary plasmid encoding the p19, a silencing suppressor protein derived from Tomato bushy stunt virus (Voinnet et al., 2003) (TBSV; kindly provided by Ulrich Commandeur, RWTH Aachen) inhibiting interfering RNA and induced with 0.2 mM acetosyringon followed by 1 h incubation at room temperature (RT). The bacteria solution was transient infiltrated to whole *N. benthamiana* plants using a desiccator. Afterward, the plants were cultivated at RT with 24 h lighting (30 μmol m^2^ s^–1^) for 5 days at high humidity supplied by a sprinkler system.

### Extraction and Purification of S1-His Antigen

For extraction and purification of the recombinant SARS-CoV-2 S1-His antigen, leaves were harvested 5 days after infiltration, and total weight was determined. Extraction of soluble proteins from the leaf material was done using an Angel juicer 8500 S (Angel Juicer Co., Ltd., Busan, South Korea). The obtained plant extract was threefold diluted by adding phosphate-buffered saline (PBS). PBS was prepared as described in Pietschmann et al. (2020a). Subsequently, the pH of the plant extract was adjusted to pH 7.4 with 1 M Tris; afterward, conductivity was set to 20 mS using a 1 M NaCl stock solution. Clarification of extract was done by centrifugation at 15,900 × *g* for 30 min using an Avanti J-26 XPI (Beckman Coulter Life Sciences, Krefeld, Germany). A final filtration step was performed before immobilized metal affinity chromatography (IMAC) purification using a Nalgene Rapid-Flow 0.2 μm filter (Fisher Scientific GmbH, Schwerte, Germany).

Cleared supernatant was subjected to IMAC protein purification using a column volume (CV) of 10 ml Chelating Sepharose Fast Flow (GE Healthcare, Uppsala, Sweden) charged with nickel ions (Ni–SO_4_, 200 mM) according to manufacturer’s instructions. The column (XK16, GE Healthcare, Solingen, Germany) was equilibrated with 5 CV PBS (pH 7.8) supplemented with 5 mM imidazole before applying the plant extract, followed by washing of bound protein with 10 CV 5 mM imidazole-supplemented PBS. Elution of bound protein was done using 4 CV PBS (pH 8.0) supplemented with 250 mM imidazole. Eluted proteins were collected in 5 ml fractions and afterward analyzed for target protein content using sodium dodecyl sulfate–polyacrylamide gel electrophoresis (SDS-PAGE). Equilibration, sample application, wash, and elution were done with a constant flow rate of 7.5 ml min^–1^.

### SDS-PAGE and Western Blot Analysis

Characterization of the recombinant SARS-CoV-2 S1 protein with respect to size, quantity, and functionality was achieved by SDS-PAGE as well as Western blot analysis as described in Voepel et al. (2014). In short, purified SARS-CoV-2 S1-His protein was separated under non-reducing and reducing conditions using 4–12% NuPAGE polyacrylamide gradient gels (Thermo Fisher Scientific, Waltham, MA, United States) and stained using standard Coomassie brilliant blue staining procedure. For Western blot analysis, proteins were transferred onto nitrocellulose membranes (Whatman, Dassel, Germany), and membranes were blocked with 5% (*w*/*v*) skim milk (Carl Roth, Karlsruhe, Germany) overnight at 4°C. Afterward, blocked membranes were washed by applying three times 0.05% (*v*/*v*) Tween-20 (Carl Roth, Karlsruhe, Germany) supplemented PBS. Then, for confirmation of appropriate antigen size or antigen functionality, His-tag targeting rabbit antibody (product number PA1-983B; Thermo Fisher Scientific, Waltham, MA, United States) diluted 1:5,000 in PBS or patient serum samples diluted 1:1,000 in PBS, respectively, was applied and incubated for 1 h at RT. Following another washing step as described above, anti-His antibody was detected with peroxidase-conjugated goat anti-rabbit antibody (product number 111-055-144; Jackson ImmunoResearch Europe Ltd., United Kingdom) diluted 1:5,000 in PBS. Visualization of bound secondary antibody was done by application of Nitro Blue tetrazolium/5-bromo-4-chloro-3indolyl-phosphate (NBT/BCIP) substrate after final washing step. SARS-CoV-2-specific IgG from patient sera were detected using biotinylated goat anti-human IgG F(ab′)_2_ fragment-specific antibody (GaH; product number 109-005-097; Jackson ImmunoResearch Europe Ltd., United Kingdom), diluted 1:5,000 in PBS followed by incubation for 1 h at RT. Biotinylation was done as described in Pietschmann et al. (2020a) using EZ-Link NHS-PEG4 Biotinylation Kit (Merck KGaA, Darmstadt, Germany). Following washing and application of peroxidase-labeled streptavidin, diluted 1:5,000, and incubation for 1 h at RT and a final washing, colorimetric detection was done as described above.

### Liaison XL-Based SARS-CoV-2-Specific Antibody Detection in Human Serum

A large set of SARS-CoV-2 positive and negative sera from patients, hospitalized in the University Hospital RWTH Aachen, were tested for the occurrence of SARS-CoV-2-specific IgG antibodies using the Liaison SARS-CoV-2 S1/S2 IgG test kit (DiaSorin, Italy) following the manufacturer’s instructions with the DiaSorin Liaison XL fully automated chemiluminescence analyzer. In brief, magnetic particles, functionalized with subunit S1 and S2 of the SARS-CoV-2 spike protein, represent the solid phase throughout the assay. By incubating patient sera on the solid phase, antigen-specific antibodies are captured. After washing of solid phase and thus removing unbound material, human IgG-specific mouse monoclonal antibody conjugated to an isoluminol derivate binds specifically onto captured patient’s antibodies. Finally, isoluminol derivate reacts after washing with the applied starter reagent, resulting in a chemiluminescence reaction. The induced signal correlates with the amount of S1- and S2-specific antibodies within patient’s serum.

### Preparation of Immunofiltration Columns

For detection of SARS-CoV-2-specific antibodies in serum specimens using magnetic immunodetection, ABICAP immunofiltration columns (IFCs, acquired from Senova Gesellschaft für Biowissenschaft und Technik GmbH, Weimar, Germany) were equilibrated as described in Rettcher et al. (2015) and Pietschmann et al. (2020a) with extended degassing time of 1 h. Preparation of equilibrated IFCs was done by coating the polyethylene matrix with 500 μl of 5 μg ml^–1^ SARS-CoV-2 S1 recombinant antigen, diluted in coupling buffer. Coupling buffer was prepared as described in Pietschmann et al. (2020a). After incubation for 1 h at RT, columns were washed with 750 μl PBS and blocked by rinsing 750 μl 5% (*w*/*v*) skim milk through IFCs by gravity flow. After incubation for additional 1 h at RT, IFCs were washed again by subsequent application of twice 750 μl PBS. Ready-to-use IFCs (RTU-IFC) can then be stored in PBS at 4°C for at least several days.

### MID-Based SARS-CoV-2-Specific Antibody Detection in Human Serum

Serum samples were 40-fold prediluted in PBS, and 400 μl of mixture was applied onto prepared and ready-to-use IFC. While diluted serum flows through IFC by gravity flow, SARS-CoV-2 S1-specific antibodies were enriched within the matrix. After incubation of 5 min at RT, a 2-min washing step with 750 μl PBS was performed prior to application of 500 μl biotinylated goat anti human (GaH), diluted 1:5,000 in PBS and subsequent incubation for 5 min. Afterward, IFCs were washed as described above, and 500 μl of 60 μg ml^–1^ streptavidin-functionalized superparamagnetic particles with a hydrodynamic diameter of 70 nm (synomag^®^-D, article number 104-19-701; micromod Partikeltechnologie GmbH, Rostock, Germany) was added to columns and incubated for additional 5 min at RT. After final washing with 750 μl PBS, measuring signal correlating to enriched S1-specific antibodies was obtained by inserting the columns into the handheld and portable frequency mixing magnetic detection device (Rettcher et al., 2015; Pietschmann et al., 2020a,b).

### MID-Based SARS-CoV-2-Specific Antibody Titer Determination in Human Serum

For the determination of SARS-CoV-2-specific antibody titer levels in human serum, positively and negatively tested sera were serially diluted starting from 20-fold dilution up to 2,560-fold dilution with PBS. After application of 400 μl diluted sera onto RTU-IFC, the assay was performed as previously described.

### Frequency Magnetic Mixing Detection Technology

Quantification of SARS-CoV-2-specific antibodies was done by sensing superparamagnetic particles within a magnetic field consisting of two distinct magnetic frequencies generated by a custom-made magnetic reader composed of two excitation coils and two measuring coils, as described in detail in Pietschmann et al. (2020a). These distinct frequencies are a high frequency magnetic field of approximately 1 mT at *f*_1_ = 49 kHz and a low-frequency field of about 10 mT at *f*_2_ = 61 Hz. Based on nonlinear superparamagnetic magnetization of streptavidin-functionalized magnetic particles, intermodulation products are generated and sensed by the detection coil. Finally, the dominant mixing component at frequency *f*_1_ + 2^⋅^*f*_2_ is demodulated. Due to the concentration dependent amplitude, magnetic particles can be detected and quantified.

### Data Analysis

All data analysis was done using GraphPad Prism 8.0.0. The threshold, defining whether a serum contains SARS-CoV-2-specific antibodies or not, was calculated using Eq. 1. Normalization of the sample’s measuring signal was done using Eq. 2. Sensitivity and specificity was calculated using Eqs. 3, 4, respectively. AIDA Image Analyzer Version 5.1 was used for quantification of separated proteins in SDS-PAGE.

(1) $$T\,h\,r\,e\,s\,h\,o\,l\,d=3*A\,v\,e\,r\,a\,g\,e_{P\,B\,S\,S\,a\,m\,p\,l\,e\,s}$$

(2) $$NormalizedMeasuringSignal=\frac{A\,m\,o\,u\,n\,t_{S\,a\,m\,p\,l\,e\,s\,p\,o\,s\,i\,t\,i\,v\,e\,t\,e\,s\,t\,e\,d\,w\,i\,t\,h\,M\,I\,D}}{A\,m\,o\,u\,n\,t_{S\,a\,m\,p\,l\,e\,s\,n\,e\,t\,a\,v\,i\,e\,t\,e\,s\,t\,e\,d\,w\,i\,t\,h\,M\,I\,D}}*100\left[\%\right]$$

(3) $$Sensitivity\left[\%\right]=\frac{A\,m\,o\,u\,n\,t_{S\,a\,m\,p\,l\,e\,s\,p\,o\,s\,i\,t\,i\,v\,e\,t\,e\,s\,t\,e\,d\,w\,i\,t\,h\,M\,I\,D}}{A\,m\,o\,u\,n\,t_{S\,a\,m\,p\,l\,e\,s\,p\,o\,s\,i\,i\,v\,e\,t\,e\,s\,t\,e\,d\,w\,i\,t\,h\,L\,i\,a\,i\,s\,o\,n\,a\,s\,s\,a\,y}}*100\left[\%\right]$$

(4) $$Specificity\left[\%\right]=\frac{A\,m\,o\,u\,n\,t_{S\,a\,m\,p\,l\,e\,s\,n\,e\,g\,a\,t\,i\,v\,e\,t\,e\,s\,t\,e\,d\,w\,i\,t\,h\,L\,i\,a\,i\,s\,o\,n\,a\,s\,s\,a\,y}}{A\,m\,o\,u\,n\,t_{S\,a\,m\,p\,l\,e\,s\,n\,e\,t\,a\,v\,i\,e\,t\,e\,s\,t\,e\,d\,w\,i\,t\,h\,M\,I\,D}}*100\left[\%\right]$$

### Liaison-Based SARS-CoV-2-Specific Antibody Detection

**FIGURE 1:**
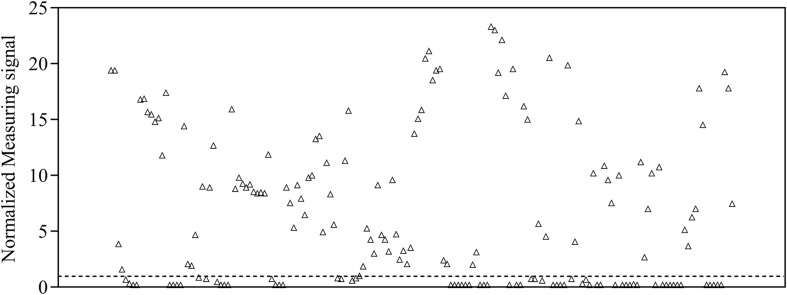
Normalized measuring signals of 170 sera samples derived from patients hospitalized in the University Hospital RWTH Aachen using DiaSorin Liaison SARS-CoV-2 S1/S2 IgG test kit in clinical environment. Each data point represents one patient (*n* = 1). Dashed line indicates the threshold defining whether antibodies are present in patient’s sample or not.

To provide a reference dataset to assess assay sensitivity and specificity of magnetic immunodetection, a total of 170 sera, derived from patients hospitalized at University Hospital RWTH Aachen, were tested using FDA-certified Liaison SARS-CoV-2 S1/S2 IgG assay (Figure 1). For this, 40-fold prediluted human sera were assayed and measuringsignal normalized according to Eq. 2. Following manufacturer’s information, the diagnostic specificity is about 99% in laboratory routine analysis. Assay sensitivity is for infections > 15 days at approximately 98%. For binding of SARS-CoV-2-specific antigens, an antigen mixture composed of subunits S1 and S2 derived from spike protein produced in human cells enabling accurate folded protein structures with appropriate glycosylation is used (DiaSorin, Italy). By this, a broad range of different neutralizing human SARS-CoV-2-specific antibodies can be captured, resulting in a high sensitivity of Liaison SARS-CoV-2 IgG assay. Based on depicted sensitivity and specificity, 105 sera resulted in a measuring signal higher than the defined threshold of 15 arbitrary units (AU ml^–1^) and consequently specified as positive regarding their SARS-CoV-2 IgG status. The remaining 65 sera were defined as negative at measuring signals lower than the defined threshold of 15 AU ml^–1^. The range of arbitrary units was between 3 AU ml^–1^ for negative sera and 350 AU ml^–1^ for positive sera, resulting in normalized measuring signals between 0.2 and 23.3.

### Generation of SARS-CoV-2 S1-His Spike Protein Antigen

**FIGURE 2:**
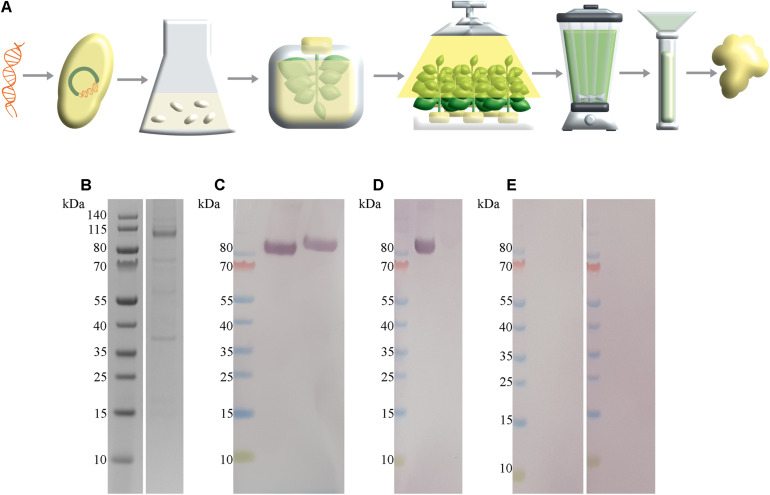
SARS-CoV-2 S1-His antigen production workflow with corresponding size and functionality analysis. **(A)** Schematic overview about plant-based transient recombinant antigen production. SARS-CoV-2 S1 protein encoding DNA was transformed into *Agrobacterium tumefaciens*. Then, *N. benthamiana* plants were vacuum infiltrated with *A. tumefaciens*. After cultivation of transiently infiltrated plants for 5 days, leaves were harvested, followed by protein extracted via juicing and purification of the target protein using immobilized metal affinity chromatography (IMAC) purification strategy. **(B)** Sodium dodecyl sulfate–polyacrylamide gel electrophoresis (SDS-PAGE) of IMAC-purified SARS-CoV-2 S1-His antigen recombinant produced in *N. benthamiana* with expected size of 80 kDa, under reducing conditions. In total, approximately 328 ng SARS-CoV-2 S1 protein was loaded. **(C)** Western blot analysis of SARS-CoV-2 S1-His protein, probed with anti-His antibody under non-reducing conditions (left) and reducing conditions (right). **(D)** Western blot analysis of 2 μg SARS-CoV-2 S1-His protein probed with patient serum previously tested positive with Liaison SARS-CoV-2 S1/S2 IgG assay and **(E)** each probed with a patient sera tested negative for the occurrence of SARS-CoV-2-specific antibodies.

To provide SARS-CoV-2 antigen protein for the development of a magnetic immunodetection-based serological assay, *N. benthamiana* leaves were coinfiltrated with *A. tumefaciens* containing SARS-CoV-2 S1-His protein encoding DNA and *A. tumefaciens* containing p19 silencing inhibitor encoding DNA. After 5 days of cultivation, plants were harvested, followed by protein extraction and purification of the target protein using IMAC. A schematic overview about recombinant plant production procedure is shown in Figure 2A. By this, a total of 2.5 mg S1-His protein at the expected size of approximately 80 kDa (non-reducing) could be obtained, as determined by separating proteins using SDS-PAGE and quantifying recombinant S1-His protein against bovine serum albumin (Figure 2B). The confirmation of appropriate antigen size was done by Western blot analysis probed with anti-His antibody revealing the expected size of approximately 80 kDa (Figure 2C). Here, antibody could bind to S1 protein in non-reduced conditions as well as in reduced conditions, confirming uniform binding capacity of SARS-CoV-2-specific IgG within human serum to recombinant generated S1-His antigen. Additionally, SARS-CoV-2 S1-His antigen was blotted and probed with one serum, previously tested positive for the occurrence of SARS-CoV-2-specific antibodies (Figure 2D), by Liaison SARS-CoV-2 S1/S2 IgG assay, and with two sera previously tested negative (Figure 2E). Here, a highly specific binding reaction of SARS-CoV-2-specific IgG against recombinant produced S1-His antigen could be observed due to the specific reaction of positive tested serum with approximately 80 kDa blotted protein. Additionally, the Western blots with in total three patient sera demonstrated the absence of cross-reactivity with remaining plant proteins within IMAC-purified protein fraction.

### SARS-CoV-2-Specific Antibody Detection Using Magnetic Immunodetection

**FIGURE 3:**
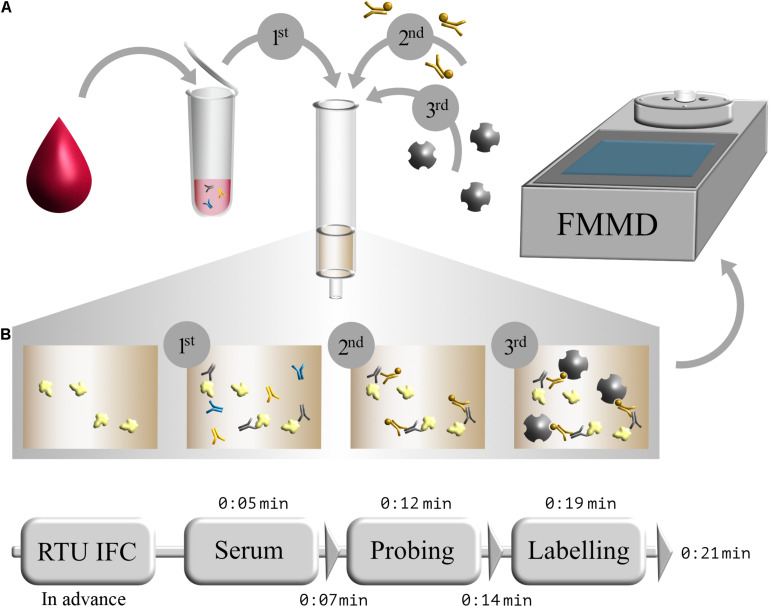
Schematic workflow of serological magnetic immunodetection for detection of SARS-CoV-2-specific antibodies in human serum. **(A)** Initial application of human blood or serum onto SARS-CoV-2 S1-antigen precoated and blocked ready-to-use immunofiltration column (IFC; 1); sample is 40-fold prediluted. Following retention of antigen-specific antibodies within the matrix, IFC is washed and loaded with biotinylated human IgG-specific secondary antibody (2). Subsequently, IFC is washed again, and streptavidin-functionalized magnetic particles were applied (3), resulting in a magnetic labeling of bound antibody complex. Finally, IFC is washed again, and enriched magnetic particles were sensed by means of frequency magnetic mixing detection (FMMD) technology. **(B)** Chronological overview of magnetic immunodetection assay duration, starting with ready-to-use immunofiltration columns (RTU-IFCs), which can be coated and blocked in advance. For analysis of SARS-CoV-2-specific antibody occurrence in human serum, prediluted serum is applied and incubated for 5 min (Serum), followed by an subsequent washing step of 2 min (depicted as triangle). Afterward, biotinylated human IgG-specific secondary antibody is applied (Probing) and incubated for additional 5 min prior another 2 min washing step. Finally, magnetic beads are applied (Labeling), incubated for 5 min and washed for 2 min, resulting in an assay duration of in total 21 min. FMMD readout takes only about 10 s.

After successful production and purification of S1-His protein, magnetic immunodetection-based SARS-CoV-2-specific antibody determination was established. Basic principle for serological SARS-CoV-2 specific IgG determination using MID is the application of serum onto S1 protein coated and blocked ready-to-use immunofiltration columns (RTU-IFCs) (Figure 3A). While the sample passes the column by gravity flow, SARS-CoV-2-specific antibodies are enriched within the matrix. Following washing, application of biotinylated human IgG-specific secondary antibody, and another washing step, streptavidin-functionalized superparamagnetic particles are applied and enrich within the matrix based on rapid and strong biotin–streptavidin interaction. Finally, magnetic particles are probed and quantified using an FMMD-based portable measuring device, whereby the measuring signal should correlate with the amount of retained specific SARS-CoV-2 antibodies within the matrix. Based on 5 min incubation and 2 min washing steps, patient’s serum can be analyzed for the presence and level of S1 protein-specific antibodies within 21 min (Figure 3B).

**FIGURE 4:**
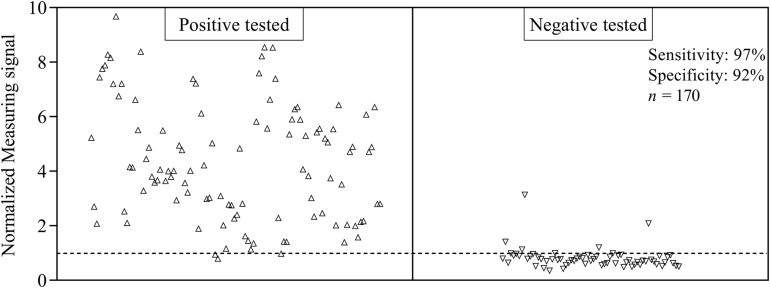
Sensitivity and specificity of magnetic immunodetection-based SARS-CoV-2-specific antibody testing. A total of 170 patient sera were previously tested with certified DiaSorin Liaison SARS-CoV-2 S1/S2 IgG assay in clinical environment for the occurrence of SARS-CoV-2-specific antibodies. Afterward, sera were retested using magnetic immunodetection with SARS-CoV-2 S1-His protein coated and blocked ready-to-use immunofiltration columns (RTU-IFCs). After application of sera, followed by subsequent addition of biotinylated human IgG-specific secondary antibody and streptavidin-functionalized magnetic particles, IFCs were measured using a handheld frequency mixing magnetic detection (FMMD) readout device. Each data point represents a patient serum (*n* = 1). Dashed line indicates the threshold defining, whether sera were positive or negative tested using magnetic immunodetection.

**FIGURE 5:**
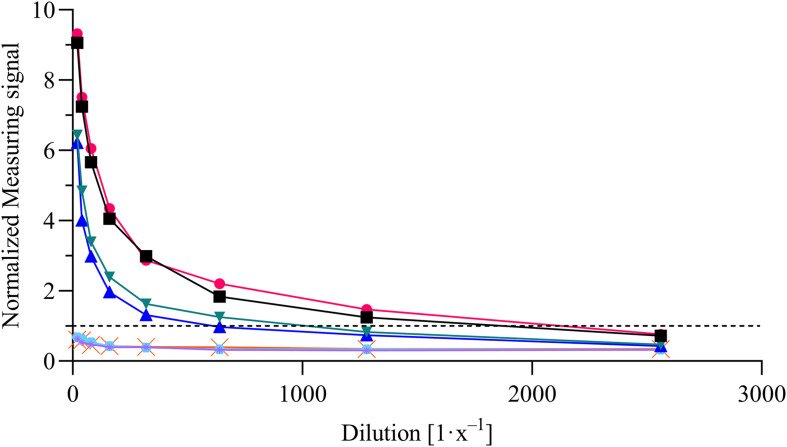
Magnetic immunodetection-based SARS-CoV-2-specific antibody titer determination. Four previously tested positive and three negatively tested patient sera were analyzed in serial dilution starting from 20-fold up to 2560-fold dilution in phosphate-buffered saline (PBS). Samples were applied onto ready-to-use immunofiltration columns (RTU-IFCs) followed by application of biotinylated anti-human IgG-specific secondary antibody. Finally, magnetic particles functionalized with streptavidin were applied, and measuring signal was determined using portable frequency mixing magnetic detection (FMMD) readout device. Each data point represents *n* = 1. Dashed line represents the threshold.

**FIGURE 6:**
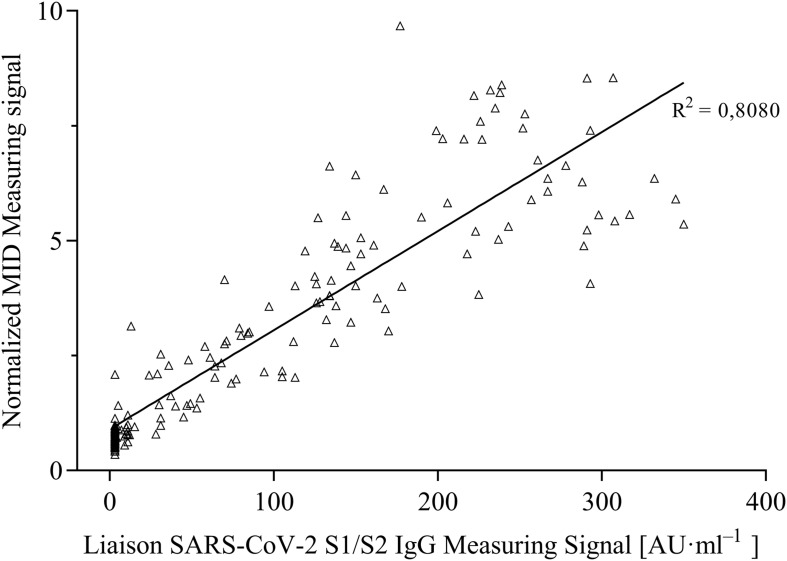
Correlation of measuring signals of certified DiaSorin Liaison SARS-CoV-2 S1/S2 IgG assay and developed portable magnetic immunodetection assay (*n* = 1).

For the evaluation of assay sensitivity and specificity, a total of 170 sera, previously analyzed using Liaison SARS-CoV-2 S1/S2 IgG assay in a clinical environment consisting of 105 positively and 65 negatively tested sera (Figure 1), were 40-fold diluted and tested on S1-His coated and blocked RTU-IFCs (Figure 4). Here, measuring signals ranging from 25.2 mV up to 849.2 mV were detected, demonstrating a broad range of detection enabling subsequent titer estimation. For visualization, measuring signals were normalized using Eq. 2. From 105 positively tested sera, 102 tested positive with MID. Three sera resulted in a measuring signal lower than calculated threshold (Eq. 1) and were defined as negative regarding the occurrence of SARS-CoV-2-specific antibodies although positively tested using Liaison assay. Following this, an assay sensitivity of 97% in comparison to the Liaison assay could be reached (Eq. 3). By using MID, 60 of previously 65 negatively tested samples generated a measuring signal below calculated threshold, resulting in five samples, which were defined as positive for the occurrence of S1-protein-specific antibodies. From those five possibly false-positive tested samples, three were factor 1.4 up to 3.4 above the threshold, whereas two were just slightly above the threshold with a factor of 1.1 or 1.2, respectively. By this, a specificity for detection of SARS-CoV-2-specific antibodies using MID of 92% could be calculated (Eq. 4). To test the possibility of SARS-CoV-2-specific antibody titer determination, four sera samples previously tested positive and three negatively tested sera were serially diluted starting from 20-fold dilution in PBS and analyzed using MID-based assay (Figure 5). Typically, the antibody titer is defined by the highest serum dilution resulting in measuring signals slightly above calculated threshold. By means of serially diluting sera samples, antibody-dose-dependent measuring signals and finally different antibody titers could be determined. Here, sera samples generating comparable measuring signals at the starting dilution finally result in quite comparable antibody titer levels. Furthermore, sera generating higher measuring signals in 20-fold dilution results in higher antibody titers than sera with lower measuring signals at 20-fold dilution. Negatively tested sera still tested negative in any dilution, thus indicating no influence of matrix interference. The dilution based on this, a correlation between antibody levels within serum sample, measuring signal and determined antibody titer can be postulated. Comparing the measuring signals of the previously performed Liaison-based SARS-CoV-2-specific antibody detection and the magnetic immunodetection-based antibody assay, a good correlation between the two assays was observed (Figure 6). Especially in the range up to 150 AU, a quite precise correlation can be seen, whereas higher measuring signals result in a larger spread of data points. However, an R^2^ of approximately 0.81 indicates a comparable assay outcome of Liaison- and MID-based antibody testing.

## Discussion

Within the context of this study, we developed a magnetic immunodetection-based serological assay for the determination of SARS-CoV-2-specific antibodies in patient sera using a plant-produced spike-protein-derived antigen. Additionally, we evaluated our assay sensitivity and specificity through the analysis of 170 patient sera, in comparison to results obtained with FDA-approved Liaison SARS-CoV-2 S1/S2 assay. While the Liaison assay is laboratory based and requires large and expensive equipment, we present a portable and fast on-site assay approach with an almost comparable assay sensitivity of 97% (based on Liaison’s assay sensitivity). Using plant-based recombinant S1 spike protein, we could reach an assay specificity of about 92% (based on Liaison’s assay specificity), whereby two of five divergent sera exceeded the threshold only very slightly. A possible reason for the observed deviations from the reference assay could be the nature of the used antigen. Recombinant proteins produced in different expression systems (mammalian cell culture for Liaison in comparison to *N. benthamiana* in our case) may differ in folding as well as host-specific differences in glycosylation patterns, which may lead to altered epitope presentation and finally differences in sensitivity and specificity (Caramelo and Parodi, 2008; Strasser, 2016). However, during development, a reduction in false-positive results could be achieved by optimizing the antigen density, and further optimizations will be attempted in future research. Nevertheless, the robust correlation between the two assays (Figure 4), as well as the high specificity and sensitivity using magnetic immunodetection (Figure 3), convincingly show that the newly developed serological MID assay is a great tool for quick and easy on-site testing of existing immunity in populations.

Especially for rapid monitoring of vaccination efficiency, a fast, easy-to-use and portable assay system might be of great interest for service providers in the medical field, for identifying immunity in elderly peoples’ and nursing homes as well as for testing people at airports and border crossings. Requiring only a single pipette and the RTU-IFC in combination with the handheld FMMD device, the assay can be performed directly on-site at various locations. Due to the simplicity of the serological magnetic immunodetection, no special know-how or training is required, and it can be handled by almost anybody after a brief introduction to the procedure. A major advantage of the developed serological magnetic immunodetection compared to the current FDA-approved LFD-based assays is that a SARS-CoV-2-specific antibody titer evaluation can be performed rapidly, and an estimation of the amount of antibody in serum can be achieved solely by the resulting measurement signal at 40-fold assay dilution. For an absolute quantitative determination of the antibody amount, a calibration measurement similar to Pietschmann et al. (2020c) or, alternatively, an antibody titer determination needs to be performed. However, the data reflect the SARS-CoV-2-specific immune status of the tested individual and can be used for the identification of previously infected persons as well as for monitoring of vaccine efficiency over time, to develop adaptive prime-boost regimens, especially in cases when there is a scarcity of vaccination doses. Currently, titer estimations can only be done using laboratory-based serological methods such as Liaison SARS-CoV-2 IgG chemiluminescence immunoassay.

Further research has to be done focusing on the assay specificity. Here, testing cross-reactivity against used SARS-CoV-2 S1-His protein from antibodies specific to other human coronaviruses (hCoVs), such as hCoV-229E, hCoV-NL63, hCoV-OC43, hCoV-HKU1, SARS-CoV, and MERS-CoV, will further increase reliability of the developed assay. Additionally, validation of specificity makes comparison to an FDA-cleared assay unnecessary. A further focus of future research is the detection of different antibody isotypes, which could be enabled by simply using other human antibody-specific secondary antibodies. By this, the course of infection could be visualized. A further approach will be the simultaneous detection of different subtypes of antibodies within the patient’s serum by using a multiplex approach as described in the preprint by Pietschmann et al. (2020c). By using frequency scanning in FMMD technology, different types of magnetic particles can be detected, which can bind differently labeled to specific isotypes of human antibodies. With this approach, the determination of infection progression would be simplified and the duration of testing procedure shortened (Achtsnicht et al., 2019b).

Additionally, the detection of viral antigen or even whole viral particles will be addressed, by using either a sandwich-based magnetic immunodetection approach or a competitive assay approach. For the sandwich-based approach, which is comparable to currently used sandwich-based LFD assays, a pair of antibodies could be used, whereby spike-protein-specific antibody is coated onto polyethylene matrix, capturing viral proteins or even whole viral particles. The second antibody, tagged with biotin, is used for enrichment of magnetic particles after binding to captured viral antigen. However, especially with low amount of viral antigen in the sample, possible false-negative results can be obtained due to low amount of bound secondary antibody, finally resulting in limited sensitivity. In a competitive setting, human ACE2 receptor could be implemented and coated onto the matrix, naturally binding spike protein of SARS-CoV-2 (Hoffmann et al., 2020; Wang Q. et al., 2020; Zhou et al., 2020). The receptor could also be recombinant produced using *A. tumefaciens*-mediated transient expression in *N. benthamiana*. For competitive reaction, the recombinant produced S1-His protein antigen can be biotinylated and thereby be used for enrichment of magnetic particles within IFC. S1-His antigen will compete for binding onto receptor with viral particles within patients sample such as saliva, resulting in a reduction in S1-His binding depending on the amount of viral antigen. By this setup, reduced but still high signals will be obtained if low amounts of viral antigen would be in the sample, resulting in a high assay sensitivity.

## Data Availability Statement

The datasets generated in this study are available from the corresponding author on request.

## Ethics Statement

The studies involving human participants were reviewed and approved by the Medical Ethics Committee of the University Hospital RWTH Aachen. The ethics committee waived the requirement of written informed consent for participation.

## Author Contributions

JP and NV: conceptualization, validation, formal analysis, investigation, and writing – original draft preparation. JP, NV, and LV: methodology. FS, HS, SR, MS, H-JK, TS, and MK: resources. JP, NV, and MK: data curation. HS, FS, H-JK, MS, LV, SR, TS, and MK: writing – review and editing. JP, NV, and HS: visualization. SR, MS, HS, and FS: supervision and project administration. All authors contributed to the article and approved the submitted version.

## Conflict of Interest

TS was employed by the company Cape Biologix Technologies. The remaining authors declare that the research was conducted in the absence of any commercial or financial relationships that could be construed as a potential conflict of interest.

## Abbreviations

CV: column volume
FMMD: frequency magnetic mixing detection
IFC: immunofiltration column
LFD: lateral flow device
MID: magnetic immunodetection
RTU-IFC: ready-to-use immunofiltration columns.
